# Superficial CD34-Positive Fibroblastic Tumor: A Newly Recognized Entity With Overlapping Features of PRDM10-Rearranged Soft Tissue Tumors

**DOI:** 10.7759/cureus.47831

**Published:** 2023-10-27

**Authors:** Nada Shaker, Rafat Abu Shakra, Omar P Sangueza

**Affiliations:** 1 Pathology, The Ohio State University Wexner Medical Center, Columbus, USA; 2 Pathology, International Medical Center, Jeddah, SAU; 3 Dermatopathology, Wake Forest Baptist Medical Center, Winston Salem, USA

**Keywords:** low-grade mesenchymal neoplasms, mesenchymal neoplasms, fibroblastic tumors, cd34-positive fibroblastic tumor, superficial cd34-positive fibroblastic tumor

## Abstract

Superficial CD34-positive fibroblastic tumor (SCPFT) is a recently identified, infrequent, low-grade mesenchymal neoplasm, first identified in 2014. Although it is relatively new to the field, SCPFT has been gaining prominence in surgical pathology practice because of its distinctive features. As of now, there are limited reported cases of SCPFT, with fewer than 100 instances documented in scientific literature. This distinctive blend of rarity and intriguing variability in presentation emphasizes the significance of identifying and understanding this uncommon entity, facilitating precise diagnosis and optimal management. In this article, we aimed to present a notable case of SCPFT in a male in his 20s who presented with a distinct subcutaneous mass measuring 2.4 × 1.8 cm at the medial aspect of the knee joint. The patient reported no significant medical history or trauma to the affected area. MRI of the knee showed a well-defined 2.4 × 1.8 cm subcutaneous mass with no definite communication with the underlying ligament or meniscus. The histopathological examination revealed spindle cell neoplasm arranged in intersecting fascicles, accompanied by arborizing blood vessels. Neoplastic spindle cells exhibited marked nuclear pleomorphism, and abundant and eosinophilic cytoplasm, with focal areas of granular, glassy, and lipidized cytoplasm. Nuclear pseudo inclusions and a few mitotic figures (1-2 per high-power field) were noted. Inflammatory infiltrates were identified within the neoplasm, comprising eosinophils and lymphocytes, highlighting an immune response within the tumor microenvironment. The surgical margin exhibited involvement with the tumor infiltrates, with the neoplastic cells extending into the adjacent fat tissue. This finding indicates local tumor spread and potential challenges in achieving complete resection. Immunohistochemical staining showed positive staining for CD34, corroborating the diagnosis of a CD34-positive fibroblastic tumor. Focal positive staining for pan-CK was noted. Staining for CD31, smooth muscle actin (SMA), desmin, S100, and anaplastic lymphoma kinase (ALK) was negative, supporting the diagnosis. The Ki67 proliferation index was low.

## Introduction

Superficial CD34-positive fibroblastic tumor (SCPFT) is a rare low-grade mesenchymal neoplasm that was recently recognized in 2014. To our knowledge, less than 100 cases have been reported in the literature with very few reports on the atypical presentation of SCPFT in a background of a myxoid stroma [1,2]. Most cases are diagnosed in middle-aged adults, an age range of 20-75 years with a mild male predominance. We aimed to report a rare case of SCPFT in a 25-year-old male who presented with a distinct 2.4 cm subcutaneous mass at the medial aspect of the knee joint. Microscopic examination revealed neoplastic cells composed of a sheet-like infiltrate of spindle cells with abundant, eosinophilic granular, and glassy cytoplasm with nuclear pleomorphism. Low mitotic figure activity was characteristic. The differential diagnosis of SCPFT encompasses a range of mesenchymal tumors, including myxofibrosarcoma, liposarcoma, and malignant peripheral nerve sheath tumors, among others. Distinguishing SCPFT from these entities requires careful evaluation of the histological features and immunohistochemical studies.

## Case presentation

**Figure 1:**
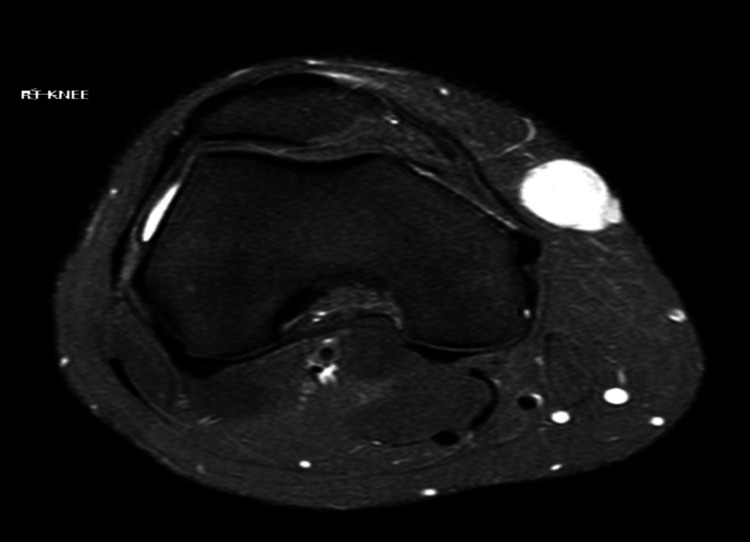
Superficial CD34-positive fibroblastic tumor: MRI of the knee. The image shows a well-defined 2.4 × 1.8 cm subcutaneous mass with no definite communication with the underlying ligament or meniscus.

A male in his 20s presented with a distinct non-painful subcutaneous mass measuring 2.4 × 1.8 cm at the medial aspect of the knee joint. The patient reported no significant medical history or trauma to the affected area. Initial clinical examination and radiographic evaluation raised suspicion for a neoplasm, necessitating further evaluation. MRI of the knee showed a well-defined 2.4 × 1.8 cm subcutaneous mass with no definite communication with the underlying ligament or meniscus (Figure 1).

**Figure 2:**
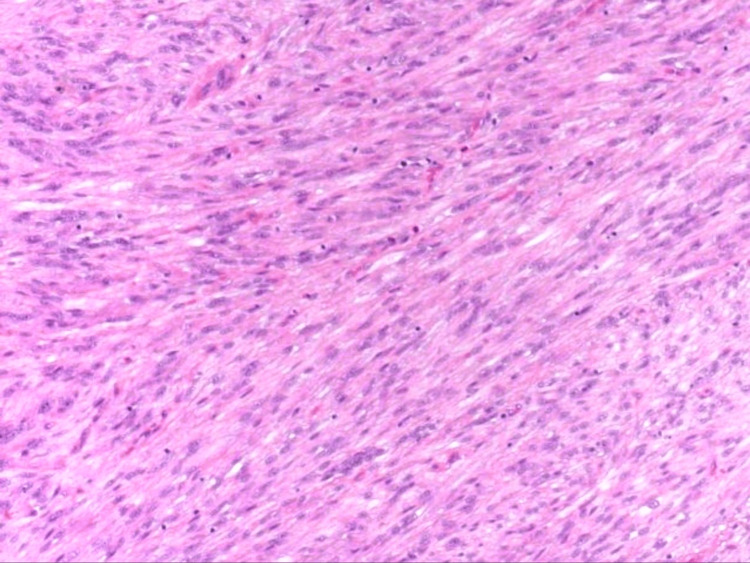
Superficial CD34-positive fibroblastic tumor: H&E staining shows spindle cell neoplasm arranged in a fascicular and whorled growth pattern. H&E stain (×100)

**Figure 3:**
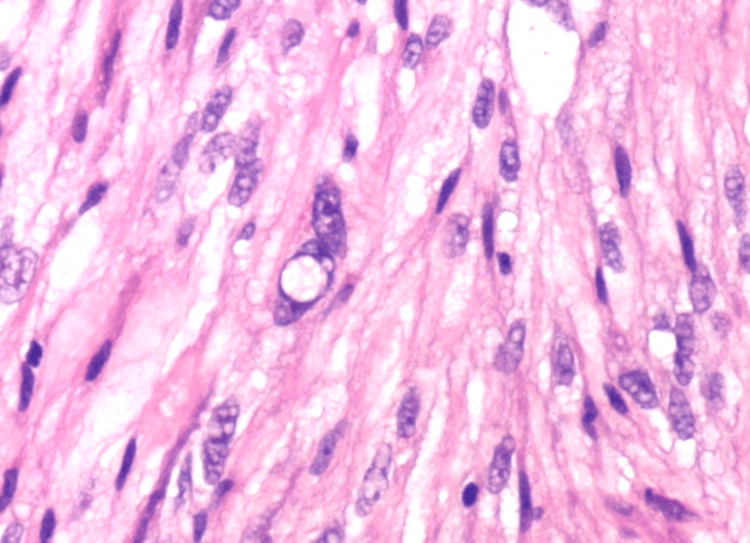
Superficial CD34-positive fibroblastic tumor: H&E staining. The image demonstrates spindle cell neoplasm with vesicular nuclei, eosinophilic glassy, and granular cytoplasm, marked nuclear pleomorphism, and nuclear pseudo inclusions.

Further evaluation with comprehensive histopathological analysis and immunohistochemical staining was warranted. The histopathological examination revealed spindle cell neoplasm arranged in intersecting fascicles, accompanied by arborizing blood vessels (Figure 2). Neoplastic spindle cells exhibited marked nuclear pleomorphism, and abundant and eosinophilic cytoplasm, with focal areas of granular, glassy, and lipidized cytoplasm (Figure 3).

**Figure 4:**
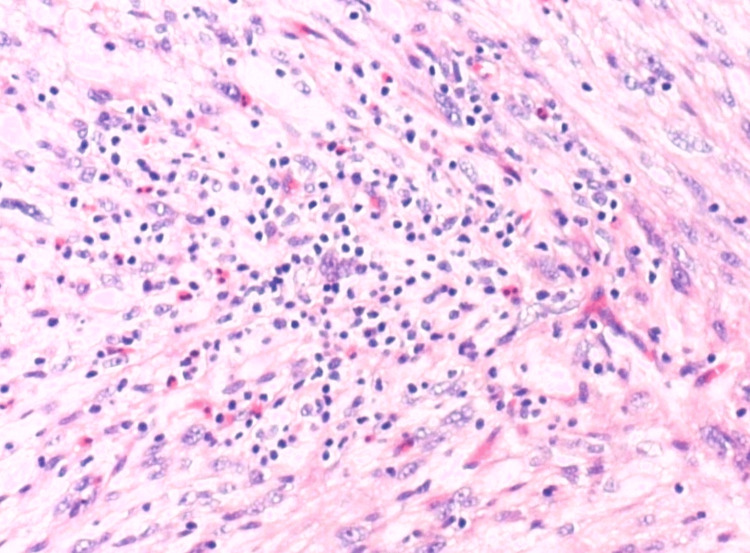
Superficial CD34-positive fibroblastic tumor: H&E staining shows inflammatory infiltrates within the neoplasm, comprising eosinophils and lymphocytes. H&E stain (×100)

Further examination revealed the presence of nuclear pseudo inclusions and a few mitotic figures (1-2 per high-power field). Inflammatory infiltrates were identified within the neoplasm, comprising eosinophils and lymphocytes, highlighting an immune response within the tumor microenvironment (Figure 4).

**Figure 5:**
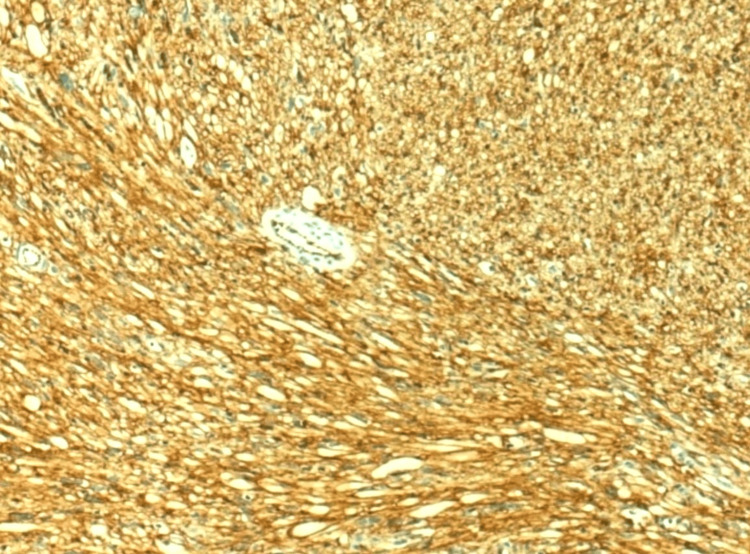
Superficial CD34-positive fibroblastic tumor: immunohistochemical staining with CD34. The image demonstrates diffuse and strong positive staining, providing additional support for the diagnosis of SCPFT (×100). SCPFT: superficial CD34-positive fibroblastic tumor

The tumor involved the surgical margin and infiltrated the adjacent fat. These findings indicated local tumor spread and potential challenges in achieving complete resection. The tumor was positive for CD34 immunohistochemical staining, corroborating the diagnosis of a CD34-positive fibroblastic tumor (Figure 5).

**Figure 6:**
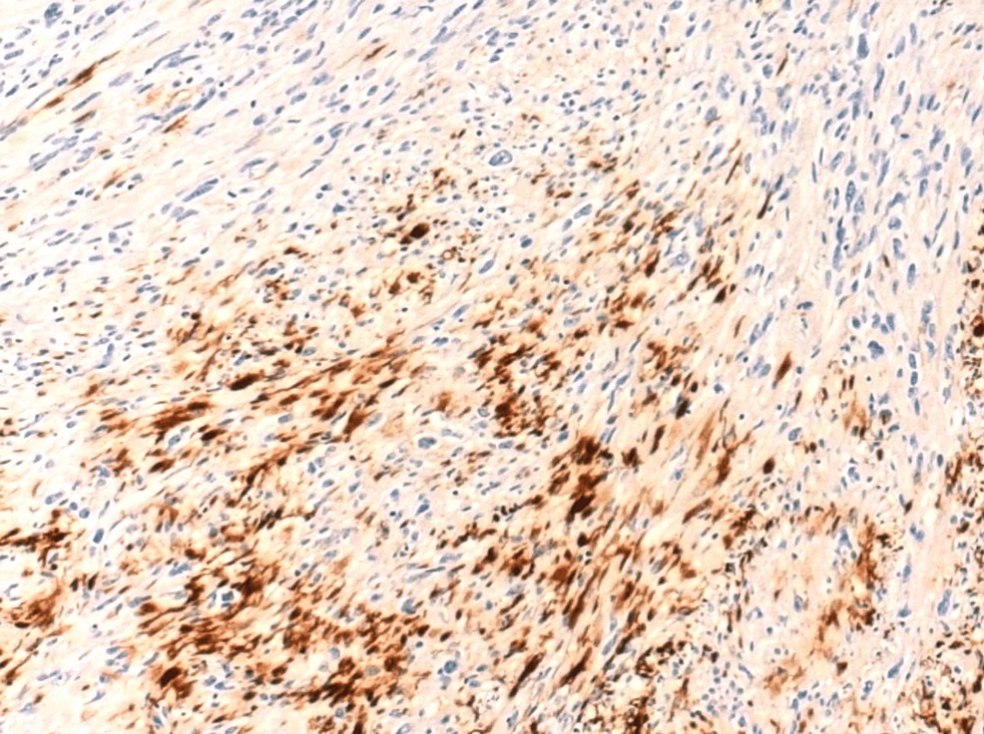
Superficial CD34-positive fibroblastic tumor: immunohistochemical staining with pan-CK. The image shows focal positive staining (×100).

**Figure 7:**
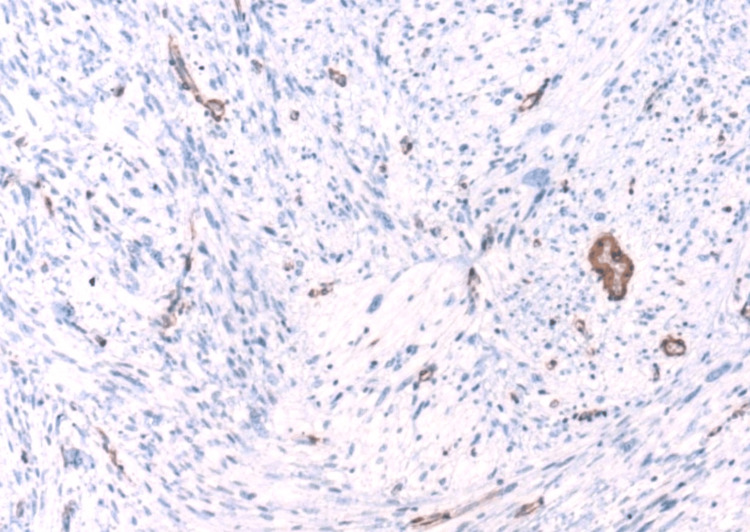
Superficial CD34-positive fibroblastic tumor: immunohistochemical staining with SMA. The image shows negative staining in tumor cells (×100). SMA: smooth muscle actin

**Figure 8:**
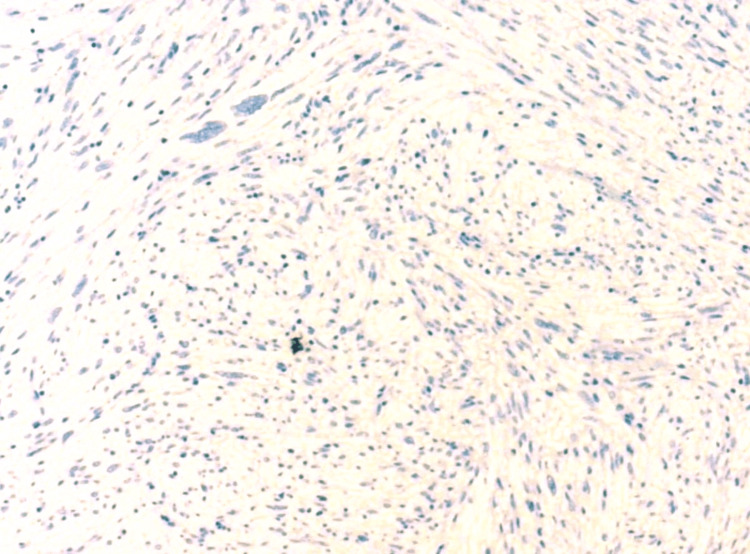
Superficial CD34-positive fibroblastic tumor: immunohistochemical staining with ALK. The image shows negative staining (×100). ALK: anaplastic lymphoma kinase

Focal positive staining for pan-CK was noted (Figure 6). Staining for CD31, smooth muscle actin (SMA), desmin, S100, and anaplastic lymphoma kinase (ALK) were negative (Figures 7, 8). The Ki67 proliferation index was low.

## Discussion

Superficial CD34-positive fibroblastic tumor (SCPFT) is a rare low-grade mesenchymal neoplasm that is rarely present in the context of the predominant myxoid stroma. Most cases exhibit characteristic presentation of neoplastic cells composed of a sheet-like infiltrate of spindle cells with abundant, eosinophilic granular and glassy cytoplasm and nuclear pleomorphism [3]. Low mitotic figure activity is often noted and characteristic. Immunohistochemical staining is essential in differentiating this entity from other mesenchymal neoplasms. Typically, spindle cells show diffuse and strong expression of CD34 with focal positive staining of cytokeratin in up to 70% of cases [4]. These features aid in distinguishing this tumor from other pleomorphic mesenchymal tumors, such as atypical fibroxanthoma and undifferentiated pleomorphic sarcoma, which are typically negative for CD34 staining.

The differential diagnosis of SCPFT encompasses a range of grade/borderline and malignant neoplasms that are superficially or deeply located tumors including atypical fibroxanthoma, myxofibrosarcoma, undifferentiated pleomorphic sarcoma, myxoinflammatory fibroblastic sarcoma, pleomorphic hyalinizing angiectatic tumor, dermatofibrosarcoma protuberans, and PRDM10-rearranged soft tissue tumors [5].

While most cases of undifferentiated pleomorphic sarcoma (UPS) are encountered deep in the subfascial region, these lesions are typically dedifferentiated and high grade with increased mitotic figures rate and necrotic debris. The lack of strong diffuse staining of CD34 expression helps in distinguishing UPS from SCPFT. In addition, UPS may exhibit recurrent PRDM10 fusions that are present in approximately 5% of cases and may indicate clinical implication [6]. Due to the high-grade feature of UPS, distant metastasis is reported in around 40% of cases [7].

Atypical fibroxanthoma (AFX) is a tumor of the elderly population that typically arises in the damaged skin of the head and neck. Notably, AFX displays a pleomorphic feature with a spindle cell variant described in the literature that might make distinguishing this entity from SCPFT challenging. However, the negative CD34 immunoreactivity aids in differentiating these two entities. The clinical behavior of AFX is benign with no infiltration of the subcutaneous fat, no necrosis, vascular or perineural invasion, and no distant metastases.

Myxoinflammatory fibroblastic sarcoma (MIFS) is a low-grade, inflammatory fibromyxoid mesenchymal tumor that frequently affects the acral soft tissue. MIFS is typically composed of atypical epithelioid cells within an inflamed fibrosclerotic stromal matrix and prominent paucicellular myxoid background and infiltrate the subcutaneous fat. Increased atypical mitotic activity is usually seen in MIFS. Diagnosis of MIFS might be challenging due to the lack of distinct morphologic, immunophenotypic, and molecular profiles. Most cases are positive for D2-40 staining. CD34 immunoreactivity is encountered in 50% of cases with keratin expression in up to a third of cases. In addition, the t (1;10) (p22; q24) TGFBR3/MGEA5 translocation is described in some cases [8]. Laskin et al. reported an overall recurrence rate of approximately 51% in a study of 104 cases of MIFS [9].

Myxofibrosarcoma (MFS) is a malignant fibroblastic neoplasm with myxoid stroma, nuclear pleomorphism, and curvilinear thin-walled blood vessels [10]. The molecular pathogenesis of MFS is complex and poorly defined. Cases of MFS show various aberrations including amplifications, deletions, and loss of function [11]. Increased mitotic activity is seen in most cases. CD34 may be patchy with absent expression for cytokeratin. Local recurrence is common, and cases with high-grade features tend to develop distant metastases [12].

IHC staining and molecular testing help in differentiating dermatofibrosarcoma protuberans (DFSP) from SCPFT. The loss of CD34 and cytokeratin expression in addition to the t(17;22) (q22;q13) translocation with COL1A1-PDGFB fusion is characteristic of DFSP [13].

Recent studies suggested that PRDM10-rearranged soft tissue tumors and superficial CD34-positive fibroblastic tumors are considered as a single entity. In general, PRDM10-rearranged soft tissue tumors have vesicular cytoplasm, low mitotic activity, and positive staining for CD34. Other types of PRDM10-rearranged soft tissue tumors may be poorly defined and display pleomorphic nuclear features within a fibrous and myxoid stroma and a prominent inflammatory infiltrate which helps differentiate these tumors from SCPFT [14].

Pleomorphic hyalinizing angiectatic tumor is a rare entity that is recently recognized as a "neoplasm of uncertain behavior" by the World Health Organization (2020) [15]. It is characterized by the presence of ecstatic blood vessels surrounded by perivascular hyaline material with potential morphological and genetic overlaps with other mesenchymal tumors such as hemosiderosis fibrolipomatous tumor (HFLT) and MIFS. The lack of keratin expression aids in distinguishing this entity from SCPFT. A t (1;10) (p22; q24) TGFBR3/MGEA5 translocation has been described in these tumors.

Most described cases of SCPFT exhibit an indolent clinical behavior with rare disease recurrence [16]. Distant metastasis is rarely reported in the literature. Therefore, this tumor is considered a low-grade mesenchymal neoplasm with borderline malignancy [17].

## Conclusions

In conclusion, superficial CD34-positive fibroblastic tumor (SCPFT) represents a rare and recently recognized entity within the realm of mesenchymal neoplasms. This relatively recent discovery, dating back to 2014, has sparked growing interest within the field of surgical pathology due to its unique and distinct characteristics.
